# Interleukin 18 Gene Promoter Polymorphisms and Susceptibility to Chronic Hepatitis B Infection: A Review Study

**DOI:** 10.5812/hepatmon.19879

**Published:** 2014-07-01

**Authors:** Mahsa Motavaf, Saeid Safari, Seyed Moayed Alavian

**Affiliations:** 1 Department of Molecular Hepatology, Middle East Liver Disease Center (MELD), Tehran, IR Iran; 2 Department of Anesthesiology and Pain Medicine, Tehran University of Medical Sciences, Tehran, IR Iran; 3 Baqiyatallah Research Center for Gastroenterology and Liver Diseases, Baqiyatallah University of Medical Sciences, Tehran, IR Iran

**Keywords:** Chronic Hepatitis B, Interleukin-18, Single Nucleotide Polymorphism, Promoter

## Abstract

**Context:**

The variation in clinical outcome of hepatitis B virus (HBV) infection is determined by virological, immunological and host genetic factors. Genes encoding cytokines are one of the candidates among host genetic factors. Polymorphisms in gene promoter can lead to different levels of cytokine expression and unique immune response. Being involved in the inflammatory cytokine network, interleukin-18 (IL-18) plays an important role in pathogenesis of HBV infection. The aim of this review is considering available literature on the association between IL-18 gene promoter single nucleotide polymorphisms (-137 C/G and -607 A/C) and susceptibility to chronic HBV infection.

**Evidence Acquisition:**

Published literature from PubMed, EMBASE, and other databases were retrieved. All studies investigating the association of IL-18 gene promoter SNPs, -137 C/G and -607 A/C, with susceptibility to chronic HBV infection were included.

**Results:**

Findings showed that the genotype -607A/A is associated with the susceptibility to chronic hepatitis B. Furthermore, allele C at position -137 is suggested to play a protective role against development of chronic HBV infection.

**Conclusions:**

Host genetic factors play an important role in determining the outcome of HBV infection. It is suggested that IL-18 genotype -607 A/A is associated with susceptibility to chronic hepatitis B. Furthermore, the carriage of allele C at position -137 may play a protective role in the development of chronic HBV infection.

## 1. Context

Hepatitis B is a potentially life-threatening liver infection caused by the HBV. It is estimated that over 2 billion of the world's population have been exposed to this virus (1-3). HBV infected patients are generally classified into one of the following clinical types:

1. asymptomatic HBV carriers;
2. acute hepatitis;
3. chronic hepatitis;
4. liver cirrhosis; and
5. primary hepatocellular carcinoma.

The reasons for variation in the pattern and clinical outcome of HBV infection are not fully understood, but are related to environmental, virological (viral load and virus genotype), immunological (host innate and adaptive immune responses) and the host genetic factors. Even though to date, no single allele has been clearly associated with HBV infection outcome or disease susceptibility, knowledge of understanding human genetic factors may provide critical clues to the ethnic diversity of HBV infection. The human genome project has indicated that there are approximately thirty-five thousand genes in the human genome. Many of the alleles contain polymorphisms such as SNPs within the encoding or non-encoding flanking regions. This high number of SNPs is likely to explain much of the genetic diversity of individuals and ethnic groups (4, 5). If a specific SNP version is found to be associated with a favorable outcome and decreased risk of progression of HBV infection and liver disease, the allele maybe considered an ‘HBV resistant’ allele. Conversely, if a version of the SNP is found to be associated with an unwanted HBV phenotype (quick disease progression or high risk of severe infection) maybe called a ‘HBV susceptible’ allele. Many recent studies are focusing on the identification for these alleles.

Most of the candidate genes fall into the following categories:

1. genes that mediate the processes of viral entry into hepatocytes, including genes involved in viral binding, fusion with cellular membrane and entrance in target cells;
2. genes that participate in the pathological alterations in liver tissue and infection type;
3. genes that modulate or regulate the immune response to HBV;
4. genes involved in the development of liver cirrhosis and hepatocellular carcinoma associated with chronic HBV infection, including genes related to mother-to-infant transmission of HBV infection; and
5. genes that contribute to resistance to antiviral therapies (6).

Host immunological response - including levels of antibodies, cytokines or cell-mediated response - plays an important role in determining susceptibility of natural course of HBV infection. Studies on SNPs within genes involved in immune responses are among those polymorphisms in various genes, such as human leukocyte antigen, tumor necrosis factor alpha (TNF-α), interferon gamma (IFN-γ), cytotoxic T lymphocyte-associated protein 4 and chemokine receptor 5 (CCR5). Many of these candidate genes polymorphisms are reported to be associated with susceptibility to chronic hepatitis B (7). 

Cytokines represent a large family of molecules, including the chemokines, interleukins, interferons and members of the TNF family, which all play an important role for the initiation and regulation of immune responses. Findings show that the capacity of cytokine production in individuals has a major genetic component (8). The remarkable differences among individuals in terms of their ability to produce cytokines have been attributed to polymorphisms within the regulatory regions of the cytokine genes. Several studies have investigated genetic polymorphisms for interleukins to identify their potential implications with respect to the natural history and treatment of viral hepatitis (9, 10). A study conducted by Li et al. (11) showed the association between IL-28B gene polymorphisms and HBV infection. In other study, Wang et al. (12) indicated the role of heterogeneity in the promoter region of the IL-10 gene in determining the initial response of chronic hepatitis B to interferon-α (IFN-α) therapy.

IL-18, also called IFN-γ inducing factor, is an obligatory cytokine for IFN-γ production and plays a key role in the induction of T-lymphocyte responses (13, 14). It is a pleiotropic pro-inflammatory cytokine that is mainly produced during the acute immune response by monocytes, macrophages, and immature dendritic cells. This cytokine also participates in both cellular and humoral responses (15). IL-18 is able to stimulate production of IFN-γ, TNF-α, IL-1, IL-2, adhesion molecules and apoptosis factors. It also increases the T-lymphocyte proliferation, and enhances the lytic activity of natural-killer cells. Considering these multiple functions, IL-18 is suggested to play an important role in the development of chronic inflammatory diseases such as chronic viral hepatitis (16). IL-18 encoding gene is located on chromosome 11q22.2–q 22.3 and consists of 6 exons and 5 introns. It lacks a TATA box, and its expression is regulated by at least two distinct promoter regions, one of which is located upstream of untranslated exon 1 (promoter 1) and the other upstream of exon 2 (promoter 2) (17, 18). 

Screening for nucleotide variations in the promoter region of this gene and being able to affect IL-18 synthesis, resulted in the discovery of several new polymorphisms (19). Two of these SNPs at positions -607 A/C and -137 G/C within the IL-18 promoter region were suggested to alter the IL-18 promoter activity (19) . G to C transversion at the position -137 and C to A transversion at the position -607 affect functionally active parts of this promoter. These parts include binding sites for histone 4 transcription factor 1 (H4TF-1) and cAMP responsive element binding protein (CREB) transcriptional, respectively. Therefore, mutation at these sites could influence IL-18 expression and alter the level of IL-18 production. So that, these two polymorphisms (-607C/A and -137G/C) and their haplotypes seem to explain difference in IL-18 expression and production (19, 20).

It has been indicated that polymorphisms in IL-18 gene promoter that alter the level of cytokine production, are association with the outcome of different infections such as hepatitis C and immune deficiency syndrome (AIDS) (21, 22). Considering these findings, recent studies investigated the possible role of the SNPs at IL -18 gene-promoter region in the progression of chronic hepatitis B.

It has been shown that IL-18 can inhibit HBV replication in the liver of transgenic mice, especially in association with IL-12 (23). Meanwhile, it has been shown that in vitro IL-18 can improve the peripheral blood monocytes (PBMC) from chronic hepatitis B patients to produce a high level of IFN-γ. These results suggested that this pro-inflammatory cytokine may contribute to the control of the HBV replication during natural HBV infection (24). Involvement of IL-18 in host immune response against HBV infection suggested that its gene polymorphisms may be linked to the outcome of hepatitis B infection. Taking this evidence into consideration, possible role of the SNPs of IL-18 gene-promoter region in the progression of chronic hepatitis B were investigated in a number of recent studies (25-27). 

## 2. Evidence Acquisition

Published literature from PubMed, EMBASE, and other databases were reviewed and all studies investigating the association of IL-18 gene promoter SNPs, -137 C/G, -607 A/C, or both with susceptibility to chronic HBV were included.

In a study by Hirankarn et al. (26) the genotype frequencies of IL-18 polymorphism at positions -607A/C between Thai patients with chronic HBV infection and healthy individuals were compared to assess whether these genes are involved in susceptibility to chronic HBV infection. The results indicated that the -607A/A genotype was significantly higher in the patients with chronic hepatitis B than in the controls. They hypothesized that -607A/A genotype likely results in markedly lower transcription activity and lower production of IL-18 from hepatic macrophage in the liver, leading to decreased inhibition of HBV replication. This hypothesis was approved by study of Khripko et al. (28) in which the authors analyzed the effect of polymorphic variants in the IL-18 gene promoter region at −607 A→C on IL-18 protein production by PBMC of healthy donors. They indicated that, the level of spontaneous IL-18 secretion in carriers of -607A/A is lower compare to C/A and C/C carriers.

In other study by Na Li et al. (27) the effect of SNP -607A/C in IL-18 gene on susceptibility to chronic HBV infection was investigated between chronic HBV infected patients and HBV natural clearance controls in Chinese population. The results showed that the genotype A/A at position -607 was present at higher frequency in patients with chronic hepatitis B compared to controls. Both studies suggested that the genotype A/A at position -607 is closely associated with the susceptibility to chronic hepatitis B and may be the susceptible gene. In contrast, a study by Zhang et al. (25) showed no significant difference in position -607 A/C gene polymorphism between patients with chronic hepatitis B and healthy controls in Chinese Han population. Ethnic and racial differences and association with other genetic markers are likely to explain these contradictory results.

Comparing genotype frequency at position -137, Zhang et al. (25) observed significant difference between the patients with chronic hepatitis B and the healthy controls in Han Chinese population. At this position, genotype G/G was present at a significantly higher frequency in the patients with chronic hepatitis B compared to those in the controls. Hirankarn et al. (26) observed the same results indicating the higher frequency of genotype -137 G/G in patients with chronic hepatitis B compared to healthy controls. However, this difference was not significant. This result does not support the hypothesis that lower production of IL-18 is associated with susceptibility to chronic HBV infection, because it is indicated that the production of IL-18 is higher in carriers of G/G compare to other genotypes (20). Investigating the distribution of allele frequency, Zhang et al. showed that the frequencies of haplotypes which bear C at position -137, in the patients with chronic hepatitis B were significantly lower than that in the healthy controls. It is in agreement with the result of Na Li et al. study (27) in which the frequency of allele C indicated to be less frequent in patients with chronic HBV compared to controls. These findings suggest that C allele at position -137 plays a protective role in the development of HBV infection. They suggested that -137 C was related with high production of IL-18, which result in better elimination of HBV.

## 3. Results

The SNPs in promoter of IL-18 gene at position -607 and -137 were suggested to have a critical impact on IL-18 gene activity, which result in different level of IL-18 production. As IL-18 plays an important role in host defense against HBV infection, difference in level of its production is suggested to be associated with HBV outcome.

Recent studies indicated that -607A/A genotype is associated by lower level of IL-18 production compare to C/A and C/C genotypes. Investigating the association between this genotype and susceptibility to development of chronic hepatitis B infection indicated a higher frequency of -607A/A in patients with chronic hepatitis B, suggesting this genotype as a susceptible gene (26, 27).

In the case of haplotype studies on -137 genotypes it has been indicated that the frequency allele C at position -137 is lower in patients with chronic HBV infection, suggesting that this allele may play a protective role in the development of chronic HBV infection (25).

## 4. Conclusions

Findings show that host genetic factors play an important role in determining the outcome of HBV infection. Because cytokines play an important role in initiation and regulation of immune responses against HBV infection, they might affect susceptibility to HBV infection. IL-18 is involved in the inflammatory cytokine network so it has an important role in the pathogenesis of various diseases including HBV infection. The SNPs in promoter of IL-18 gene at position -607 and -137 were contributed to difference in IL-18 gene activity, which result in variable level of IL-18 production. A number of recent studies have demonstrated the association between -607A/A genotype and the susceptibility to development of chronic hepatitis B (26, 27). Furthermore, the carriage of allele C at position -137 in the promoter of IL-18 gene is suggested to play a protective role against development of chronic HBV infection (25). However, the real roles of IL-18 gene promoter polymorphisms in the pathogenesis of developing chronic hepatitis B needs to be further investigated within large population from different ethnic groups and geographic regions.
